# Transgenic Cry1Ab Rice Does Not Impact Ecological Fitness and Predation of a Generalist Spider

**DOI:** 10.1371/journal.pone.0035164

**Published:** 2012-04-12

**Authors:** Jun-Ce Tian, Yang Chen, Zhao-Liang Li, Kai Li, Mao Chen, Yu-Fa Peng, Cui Hu, Anthony M. Shelton, Gong-Yin Ye

**Affiliations:** 1 State Key Laboratory of Rice Biology, Ministry of Agriculture Key Laboratory of Agricultural Entomology, Institute of Insect Sciences, Zhejiang University, Hangzhou, Zhejiang, China; 2 Department of Entomology, Cornell University/New York State Agricultural Experiment Station (NYSAES), Geneva, New York, United States of America; 3 Institute of Biological Sciences and Biotechnology, Donghua University, Shanghai, China; 4 State Key Laboratory for Biology of Plant Diseases and Insect Pests, Institute of Plant Protection, Chinese Academy of Agricultural Sciences, Beijing, China; Universidad Nacional Autonoma de Mexico, Instituto de Biotecnologia, Mexico

## Abstract

**Background:**

The commercial release of rice genetically engineered to express a Cry1Ab protein from *Bacillus thuringiensis* (Bt) for control of Lepidoptera in China is a subject of debate. One major point of the debate has focused on the ecological safety of *Bt* rice on nontarget organisms, especially predators and parasitoids that help control populations of insect pests.

**Methodology/Principal Findings:**

A tritrophic bioassay was conducted to evaluate the potential impact of Cry1Ab-expressing rice on fitness parameters of a predaceous ground spider (*Pardosa pseudoannulata* (Bösenberg et Strand)) that had fed on *Bt* rice-fed brown planthopper (*Nilaparvata lugens* (Stål)) nymphs. Survival, development time and fecundity of this spider were not different when they were fed with *Bt* rice-fed or non-*Bt* rice-fed prey. Furthermore, ELISA and PCR gut assays, as well as a functional response trial, indicated that predation by *P. pseudoannulata* was not significantly different in *Bt* rice or non-*Bt* rice fields.

**Conclusions/Significance:**

The transgenic Cry1Ab rice lines tested in this study had no adverse effects on the survival, developmental time and fecundity of *P. pseudoannulata* in the laboratory or on predation under field conditions. This suggests that this important predator would not be harmed if transgenic Cry1Ab rice were commercialized.

## Introduction

Rice, *Oryza sativa* L., is one of the most important food staples in the world. More than 50% of the world population (or more than 3 billion people) depend on rice for their daily lives [1]. However, according to various estimates, 40% more rice must be produced to meet the increasing needs of the projected human population of 2030 [2]. Genetic improvement of rice varieties through modern biotechnology to increase tolerance or resistance to biotic and abiotic stresses is one strategy to help meet the demands of the growing global populations, especially in developing countries [2]. Rice expressing Cry1Ab from the bacterium *Bacillus thuringiensis* (Bt), has been developed to control Lepidoptera, stem borers and leaffolders [3] in China. Despite the fact that no *Bt* rice lines have yet been approved for commercial release in China, a *Bt* rice variety (Huahui1) that expresses Cry1Ab and its hybrid line (Shanyou 63) have been granted biosafety certificates and approved for limited release in farmers' fields in Hubei Province from 2009 to 2014 [4].

*Bt* rice expressing Cry1Ab is on the verge of being commercially released in China, however the effects of *Bt* rice on the environment continue to be debated. Although *Bt* rice targets Lepidoptera, other insects may come into contact with the Cry1Ab protein by feeding on the plant or by feeding on insects that have fed on the plant. Thus, the potential for deleterious effects of *Bt* crops on a variety of insects need to be evaluated cautiously and systematically prior to commercialization [5], [6], [7]. Several studies of the effects of *Bt* rice on non-target arthropods have been carried out under laboratory and field conditions, but few studies have been conducted on non-target natural enemies, which play a vital role in pest control [3], [8], [9].

Spiders are common and abundant predators that suppress pest populations in many agro-ecosystems including rice [10], [11]. *Pardosa pseudoannulata* (Bösenberg et Strand) (Araneae: Lycosidae) is a dominant spider species in rice fields in China [12]. It lives in the rice paddy and is recognized as a significant biological control agent of insect pests, especially the brown planthopper, *Nilaparvata lugens* (Stål) (Hemiptera: Delphacidae), which is one of the most serious rice pests in south Asia [13], [14]. Although *N. lugens* is not susceptible to Cry1Ab [15], it is consumed by generalist spiders that are important for its control. Previous studies have examined the effects of *Bt* rice on the occurrence of five common spider species and the survival, developmental time and fecundity of two of them: *Pirata subpiraticus* (Bösenberg et Strand) ((Araneida: Lycosidae) and *Ummeliata insecticeps* (Bösenberg et Strand) (Araneida: Linyphiidae) [16], [17], [18]. However, the impact of *Bt* rice on the actual predation of important rice insect pests in the field by spiders has not been explored. In this article, we evaluated (1) biotransfer of Cry1Ab insecticidal protein in a food chain comprising two *Bt* rice lines, the herbivore *N. lugens* and the predator *P. pseudoannulata*, (2) prey-mediated effects of *Bt* rice on the survival, development, fecundity and functional response of the predator *P. pseudoannulata* under laboratory conditions, and (3) ecological impact of *Bt* rice on the predation of *P. pseudoannulata* in the field using ELISA and PCR assays.

## Results

### Cry1Ab in Bt rice, N. lugens and P. pseudoannulata

**Figure 1:**
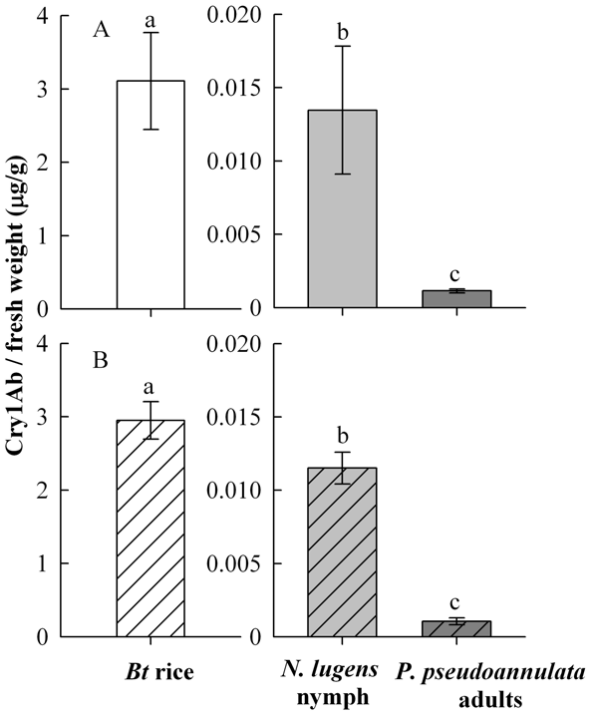
Levels of Cry1Ab protein (mean ± SE) detected from *Bt* rice and *Bt* rice-reared *Nilaparvata lugens* nymphs, and *Pardosa pseudoannulata* adults fed on *Bt* rice-fed *N. lugens* larvae using ELISA. (A) KMD1, (B) KMD2. *n* = 5 for *Bt* rice and *Nilaparvata lugens* nymphs; *n* = 3 for *Pardosa pseudoannulata* adults. Means denoted with different lowercase letters were significantly different (one-way ANOVA, *P*<0.05). Note difference in scale for Cry1Ab concentrations in plants and insects.

The concentrations of Cry1Ab detected in *Bt* rice stems, *N. lugens* and *P. pseudoannulata* in laboratory bioassays are shown in Fig. 1. In KMD1 rice stems, high concentrations of Cry1Ab were detected (2.95±0.25 µg/g of fresh weight (FW)); however, Cry1Ab concentrations in KMD1 rice-reared *N. lugens* nymphs (13.46±4.36 ng/g of FW) and *P. pseudoannulata* adults fed on KMD1 rice-reared *N. lugens* nymphs (1.14±0.14 ng/g of FW) were significantly lower. Cry1Ab concentrations in prey were significantly higher than those in predators (*F* = 338.28; df = 2, 10; *P*<0.001). Likewise, the concentrations of Cry1Ab detected in arthropods were significantly lower than those in KMD2 rice stems (2.95±0.25 µg/g of FW) (*F* = 1623.26; df = 2, 10; *P*<0.001). The average Cry1Ab concentration in KMD2 rice-reared *N. lugens* nymphs (11.67±1.45 ng/g of FW) was significantly higher than those in *P. pseudoannulata* adults fed on KMD2 rice-reared *N. lugens* nymphs (1.06±0.23 ng/g of FW). No Cry1Ab was detected in non-*Bt* controls Xiushui 11.

### Prey-mediated effects of *Bt* rice on predator survival, development and fecundity

**Figure 2:**
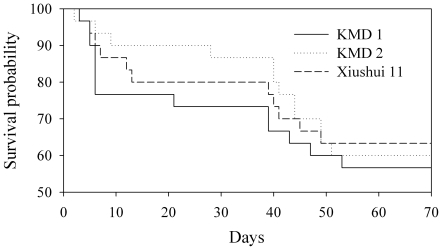
Survival of *Pardosa pseudoannulata* over a 70-day period when fed with either *Bt* rice-fed or non-*Bt* rice-fed *Nilaparvata lugens* nymphs. There was no significant difference between *Bt* rice and control treatment, based on Wilcoxon test. *n* = 60 for KMD1, KMD2 and Xiushui 11.

A tritrophic bioassay was performed to investigate the effects of *Bt* rice on *P. pseudoannulata* via dosed *N. lugens*. The results indicate that over a period of 70 days when all adults had emerged, the survival of *P. pseudoannulata* was not significantly affected when the spiders were supplied with *Bt* rice-reared (KMD1 and KMD2) *N. lugens* compared with non-*Bt* rice-reared (Xiushui 11) *N. lugens* (χ^2^ = 0.6868; df = 2; *P* = 0.71) (Fig. 2).

Furthermore, the development time (from 2nd instar to adult) of *P. pseudoannulata* fed on *Bt* rice-reared *N. lugens* was not significantly different from those that fed on non-*Bt* rice-reared *N. lugens* (*F* = 1.35; df = 2, 107; *P* = 0.267) (Table 1). However, the duration of 2nd instar spiders was longer in KMD2 than in Xiushui 11 (*F* = 3.63; df = 2, 149; *P* = 0.031), and the duration of 4th instar spiders was shorter in KMD2 than in Xiushui 11 (*F* = 2.96; df = 2, 147; *P* = 0.058). No significant difference was found in the development time for the other spiders instars.

**Table 1:** Developmental time (mean ± SE) of *Pardosa pseudoannulata* from 2nd instar to adult emergence when fed either *Bt* rice-fed (KMD1 and KMD2) or non-*Bt* rice-fed (Xiushui11) *Nilaparvata lugens*.

Development time (days)		Rice varieties	
	KMD1	KMD2	Xiushui 11
2nd instar	7.3±1.0 ab (48)	7.3±1.3 a (56)	6.6±0.8 b (56)
3rd instar	11.6±2.2 a (45)	11.2±2.4 a (54)	10.9±2.6 a (50)
4th instar	7.0±2.8 ab (44)	6.4±1.7 b (54)	8.1±2.9 a (50)
5th instar	8.0±2.2 a (42)	8.6±1.7 a (52)	8.1±2.3 a (50)
6th instar	6.8±1.6 a (38)	7.5±2.0 a (47)	7.0±2.0 a (45)
7th instar	11.2±2.8 a (34)	10.0±1.7 a (36)	11.7±3.9 a (40)
8th instar	13.7±1.7 a (18)	13.3±1.5 a (25)	14.5±1.2 a (20)
2nd instar-adult	59.4±1.2 a (34)	61.7±0.8 a (36)	60.2±0.9 a (38)

Mean (± SE) followed by different letters in the same row are significantly different (One-way ANOVA, *P*<0.05). (n), number of individuals at each development stage.

Columns do not add up to the development time for 2nd instar – adult since some *P. pseudoannulata* had six molts before they reached the adults stage and others had seven molts.

*Pardosa pseudoannulata* adults emerging from feeding trials were assigned to breeding pairs, and also fed either *Bt* rice-fed or non-*Bt* rice-fed *N. lugens*. Female spiders supplied with KMD1-, KMD2- and Xiushui11- fed *N. lugens* laid 91.8±20.9, 92.6±12.0, 95.0±16.0 eggs per sac and 86.2±6.9, 86.8±2.2, 89.9±4.9% eggs hatched, respectively. The number of eggs in the first egg-sac and the egg hatching rate of *P. pseudoannulata* were not significantly different between *Bt* and non-*Bt* rice (number of eggs: *F* = 0.05; df = 2, 29; *P* = 0.952; egg hatching rate: *F* = 0.79; df = 2, 29; *P* = 0.478).

### Effects of *Bt* rice on functional response of *P. pseudoannulata*

**Figure 3:**
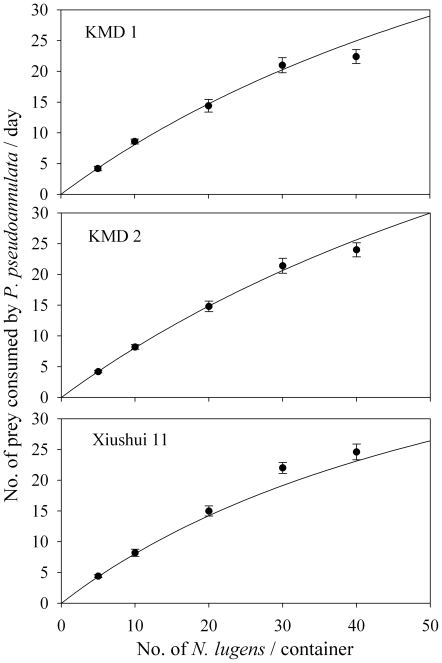
Functional response of *Pardosa pseudoannulata* collected from KMD1, KMD2 and Xiushui 11 fields.

The predator's rate of feeding upon prey, or its functional response, is one significant component in understanding the relationship between predator and prey. In 1959, Holling introduced three types of functional response that represent an increasing linear relationship (Type I), a decelerating curve (Type II), or a sigmoidal relationship (Type III) [19]. In this study, for all rice types, the functional response of *P. pseudoannulata* was typically that described as Holling Type II (Fig. 3). The instantaneous search rate and handling time, which are two important parameters of functional response, were not significantly affected by rice type (P>0.05) (Table 2).

**Table 2:** Parameters of Type II functional response by *Pardosa pseudoannulata* to *Nilaparvata lugens* from *Bt* or non-*Bt* rice fields.

Tested material	*a*	*b*	R^2^ (%)
KMD 1	1.002±0.118	0.018±0.004	92.15
KMD 2	0.965±0.096	0.015±0.003	94.47
Xiushui 11	0.972±0.094	0.014±0.003	94.76

*a*: instantaneous search rate (day^−1^).

*b*: time required to handle a prey (day).

There was no significant difference between *Bt* rice and control treatment, based on one-way ANOVA (P<0.05).

### Laboratory spider feeding trial

**Figure 4:**
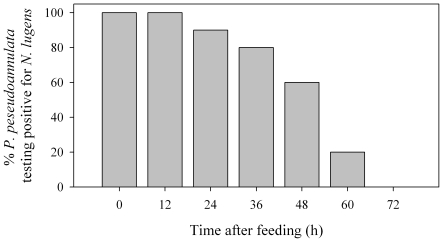
Percentage of *Pardosa pseudaoannulata* testing positive for *Nilaparyata lugens* remains 0–72 h after prey ingestion. *n* = 10 individuals per interval and 3 negative controls.

The *N. lugens*-specific primer set was used to amplify a fragment (351-bp) of COI specific to *N. lugens*. There was no cross activity between *N. lugens* and *Sogatella furcifera* (Horváth), *Laodelphax striatellus* (Fallén), *Nephotettix cincticeps* Uhler, *Chlorops oryzae* Matsumura, *Hydrellia griseola* (Fallén), *Hysteroneura setariae* (Thomas), or *Cyrtohinus lividipennis* Reuter that *P. pseudoannulata* may prey on. The polymerase chain reaction (PCR) assay was 100% effective at detecting *N. lugens*' DNA in *P. pseudoannulata* at 0 h and 12 h after the spiders fed (Fig. 4). The percentage of spiders testing positive for prey at 0, 12, 24, 36, 48, 60 and 72 h could be described using the following equation, *y* = 97.30/(1+EXP (−6.64+0.13*x)*) (*F* = 183.28; df = 2, 6; *P* = 0.0001; *R^2^* = 0.9892). The prey detection half-life for spiders was estimated at 49.8 h.

### Field study

**Figure 5:**
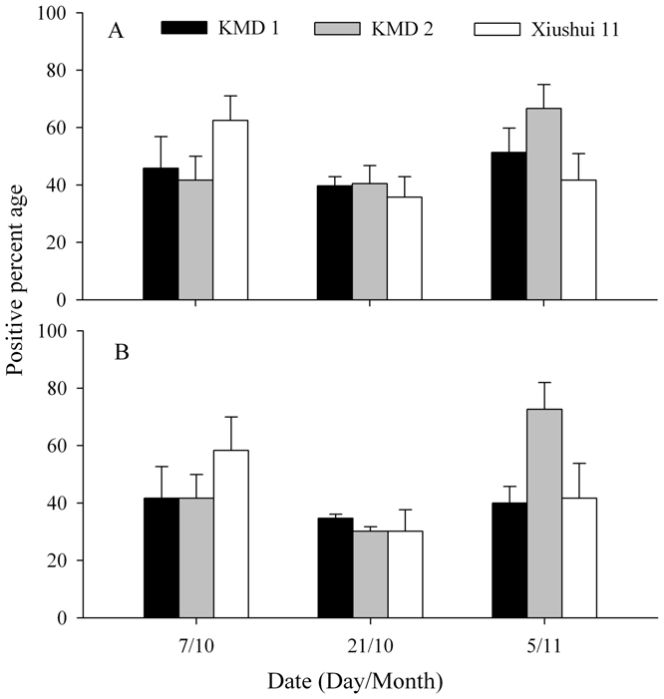
Percentage of *Pardosa pseudoannulata* testing positive for *Nilaparvata lugens* remains under field conditions using ELISA (A) and PCR (B). *n* = 3. There was no significant difference between *Bt* rice and control treatment, based on repeated-measures ANOVA (*P*<0.05).

A total of 1,620 *P. pseudoannulata* were examined for *N. lugens* remains by ELISA and PCR assay. The results are shown in Fig. 5. In ELISA assays, the percentage of spiders testing positive for *N. lugens* remains was not significantly affected by rice type (*F* = 0.17; df = 2, 50; *P* = 0.8486), sampling date (*F* = 2.37; df = 2, 50; *P* = 0.1359) and the interaction between rice type and sampling date (*F* = 1.84; df = 2, 50; *P* = 0.1869). Similarly in PCR assays, rice type (*F* = 0.85; df = 2, 50; *P* = 0.4723), sampling date (*F* = 2.24; df = 2, 50; *P* = 0.1495) and the interaction between rice type and sampling date (*F* = 1.28; df = 2, 50; *P* = 0.3312) did not significantly influence the percentage of spiders testing positive for prey.

## Discussion

On 27 November 2009, *Bt* rice and biotech phytase maize were approved to be planted in Hubei and Shangdong Provinces, respectively, by the government of China [4]. These approvals have enormous implications for biotech crop adoption not only for China and Asia, but for the whole world [20]. It is likely that China will be the first in the world to commercially produce *Bt* rice. Data on potential effects of *Bt* rice on non-target arthropods, especially insect natural enemies through tritrophic interaction, are needed prior to commercialization. However, it is not feasible to evaluate the potential risk to every species, since 46 species of predators are known to occur in rice [21]. So key species that are representative of different functional groups should be selected and potential hazards evaluated with representative surrogate/indicator species [7]. *P. pseudoannulata* is a polyphagous predator in the rice ecosystem in most of Asia and is one of the most dominant spiders. It can effectively regulate the pest populations of leafhoppers and planthoppers, and is the most important predator of the brown planthopper *N. lugens* [12], [13], [22], [23], [24], [25] and should be considered an excellent representative species of polyphagous predators. However, to date, no study has evaluated the impact of *Bt* rice on this agronomically important spider species.

In the present study, Cry1Ab protein was detected in *Bt* rice, prey and predator (Fig. 1). This indicates that Cry1Ab protein can be transferred from lower to higher trophic levels through tritrophic interactions. However, no bioaccumulation of Cry1Ab protein occurred, since the level of Cry1Ab protein detected in the predator was 11-fold lower than that in the prey. The similar dilution of the *Bt* protein through tritrophic interactions has been reported in other tritrophic experiments. For example, Torres and Ruberson demonstrated that Cry1Ac protein levels detected in predators (the big-eyed bug, *Geocoris punctipes* (Say) (Heteroptera: Lygaeldae), and the damsel bug, *Nabis roseipennis* Reuter (Hemiptera: Nabidae) were 25-fold and 7-fold lower, respectively, than that in their prey (the two-spotted spider mite, *Tetranychus urticae* Koch (Acari: Tetranychidae)) in *Bt* cotton systems [26]. Li and Romeis found the Cry3Bb1 protein concentration in the ladybird beetle, *Stethorus punctillum* Weise (Coleoptera: Coccinellidae), larvae and adults were 6-fold and 20-fold lower, respectively, than that in its spider mite prey, *Tetranychus urticae* Koch (Acari: Tetranychidae) in *Bt* maize systems [27]. Similarly, the level of Cry1Ab protein detected in the predator *Ummeliata insecticeps* (Bösenberg et Strand) (Araneida: Linyphiidae) supplied with *Bt* rice-fed prey *N. lugens* was about 5-fold lower than that in the prey in *Bt* rice systems [18].

The tritrophic experiments were carried out to evaluate the impact of *Bt* rice varieties on the biology and reproduction of *P. pseudoannulata* via its prey *N. lugens*. Results showed *Bt* rice expressing Cry1Ab (KMD1 and KMD2) did not affect survival, developmental time and fecundity of *P. pseudoannulata*. This is consistent with other laboratory studies that assessed the potential effects of *Bt* protein on spiders. No negative prey-mediated effects on two spiders, *Hylyphantes graminicola* (Sundevall) (Araneae: Linyphiidae) and *Coleosoma octomaculatum* (Böesenberg et Strand) (Araneae: Theridiidae) were observed when they fed on *Bt* cotton (expressing Cry1Ac protein)-fed prey [28]. Similarly, *Bt* maize expressing Cry3Bb1 had no adverse impact on mortality, weight development or offspring production of the web-building spider, *Theridion impressum* L. Koch (Araneae: Theridiidae) [29]. Tian et al. also reported that, although *U. insecticeps* ingested measurable amounts of Cry1Ab protein when it was supplied with *Bt* rice-fed *N. lugens*, *Bt* rice did not have negative effects on the developmental time and fecundity of *U. insecticeps* [18].

To date, no adverse effects of *Bt* crops on the biology and reproduction of spiders have been reported. However, this is not the same as evaluating predation which should also be evaluated. One method is to determine the functional response of a predator and another is to track the prey in the predator's gut. ELISA and PCR assays have been used to study predation in many studies (reviewed by [30], [31]). However, most of them, especially PCR assays, have focused more on the development of the assays than the application of the assays. To date, no study on predation has been conducted to evaluate the effect of Bt crops on predation of non-target predators in the field. In this study, we measured the predation of *P. pseudoannulata* on *N. lugens* in *Bt* rice (KMD1 and KMD 2) and non-*Bt* rice fields using ELISA and PCR gut content assays. Results indicated there was no significant difference between *Bt* rice and non-*Bt* rice fields when using both assays (Fig. 5). Fournier et al. conducted a comparative study of the efficacy of an ELISA and PCR gut content assay [32] and did not find one assay superior, but found a high degree of variability between the two assay techniques. This suggests that ELISA and PCR assays should be used together to obtain a more reliable result. Both our ELISA and PCR assays showed no significant difference between *Bt* and non-*Bt* fields which confirmed *Bt* rice does not affect the predation of *P. pseudoannulata*. More importantly, our functional response trial also indicated that *Bt* rice did not affect predation behavior. Similar results are reported by Liu et al., about *H. graminicola* raised on *Bt* cotton-fed or non-*Bt* cotton-fed *Helicoverpa armigera* having similar functional responses [28].

In conclusion, the transgenic Cry1Ab rice lines tested in this study, had no adverse effects on the survival, developmental time and fecundity of *P. pseudoannulata* in the laboratory, nor did they differ in predator functional response or on predation under field conditions. Those results, together with the published literature, suggest that spiders are not likely to be harmed by the cultivation of *Bt* rice expressing Cry1Ab and could continue playing important roles in biological control of insect pest management in rice when Cry1Ab rice is fully commercialized in China.

## Materials and Methods

### Ethics Statement

All necessary permits were obtained for the described field studies. Permission of small-filed test of the transgenic lines (KMD1 and KMD2) at the Experiment Farm of Zhejiang University in 2009 was issued by Ministry of Agriculture of the People's Republic of China. Contact: Rong Jin; Phone: +86 (571) 86971935.

### Plant materials

Transgenic rice lines, KMD1 and KMD2, were modified using an *Agrobacterium*-mediated method to transfer a *cry1Ab* gene under the control of the maize *ubiquitin* promoter. The untransformed parental commercial cultivar (Xiushui 11) was used as control. The lines, selected through fifteen generations, were homozygous for the transgenes (*cry1Ab*, *β-glucuronidase*, *neomycin phosphotransferase II*) [33].

### Insects

A *P. pseudoannulata* colony was established from the Experiment Farm of Zhejiang University (120.12°E, 30.13°N) in May 2008. Fifty pairs of spiders were collected from fields and maintained in a climatic chamber (28±2°C, 80±5% RH and L∶D 16∶8 h photoperiod). The *P. pseudoannulata* second instars were used in bioassays. A laboratory colony of *N. lugens* was provided by the China National Rice Research Institute. *Nilaparvata lugens* pairs were reared on rice plants transplanted into plastic pots and covered with a cage made from a transparent plastic cylinder (11 cm diameter, 40 cm high) with nylon mesh at the top for ventilation in a temperature-controlled room (28±2°C, 80±5% RH and L∶D 16∶8 h photoperiod). The 2nd or 3rd instar nymphs of *N. lugens* that had fed on KMD1, KMD2 and Xiushui 11 were used in bioassays.

### Bioassay of the effects of *Bt* rice on *P. pseudoannulata* via tritrophic interactions

For each treatment, 60 individual 2nd instar spiderlings of *P. pseudoannulata* were kept in separate small glass tubes (2 cm diameter, 12 cm high). The mouths of the tubes were covered with cotton balls. The bottom of each tube was filled with a piece of wetted sponge to maintain humidity. The tubes remained in a temperature-controlled room (28±2°C, 80±5% RH and L∶D 16∶8 h photoperiod). Spiders were supplied with KMD1-, KMD2-, or Xiushui 11-fed 2nd or 3rd instar *N. lugens*. The survival of *P. pseudoannulata* was monitored on a daily basis and developmental time (time to adult emergence) was also recorded. On emergence, the male and female spiders from each treatment were paired and mated. During the bioassay, the number of spiderlings hatching from the first egg sac per female was recorded as well as the number of unhatched eggs in the sac.

### Cry1Ab detection in rice plants, *N. lugens* and *P. pseudoannulata*

Rice stems, *N. lugens* nymphs and *P. pseudoannulata* adults used in the lab experiments were collected individually, transferred into 1.5 ml Eppendorf tubes and frozen at −70°C. To determine Cry1Ab protein levels in samples, ELISAs were carried out using QualiPlate Kit for Cry1Ab/Cry1Ac (Envirologix, Portland, ME). For each treatment, samples (5 for rice stems and *N. lugens* nymphs (100 mg as one replicate), 3 for *P. pseudoannulata* adults (two spiders as one replicate)) were weighed, homogenized and diluted (×500 (mg/µl) for rice stems; ×5 (mg/µl) for *N. lugens* and *P. pseudoannulata*) in phosphate-buffer saline solution in Tween20. The solution was mixed for 1 min on a vortex mixer, centrifuged for 5 min at 12,000× g, and loaded at 50 µl per test well. After dispensing 50 µl enzyme conjugate per well and incubating 2 h in a humid box at room temperature, 100 µl of the 3, 3′, 5, 5′-tetramethylbenzidine (TMB) substrate solution was added for color development. At the end of the 15-minute incubation with TMB substrate, 50 µl of 3 M sulfuric acid was added to each well. Spectrophotometric measurements were taken using a multi-detection microplate reader (Synergy HT, Bio-Tek, Winooski, VT) at 450 nm. Purified Cry1Ab (EnviroLogix, Portland, ME) toxin at concentrations of 0, 0.3125, 0.625, 1.25, 2.5, 5, 10 and 20 ng/ml were used as calibrators.

### Effects of *Bt* rice on functional response of *P. pseudoannulata*

For the functional response experiment, 25 healthy female *P. pseudoannulata* adults of similar size were collected from each plot (KMD1, KMD2 and Xiushui 11), transferred to individual glass tubes and starved for 72 h before the experiment. Each individual spider was placed in a glass jar (9 cm diameter, 10 cm high) containing four 15d-old Xiushui 11 seedlings infested with 3rd instar *N. lugens*. The prey densities used were 5, 10, 20, 30 and 40 per jar. The trial was replicated 5 times for each density. The number of *N. lugens* killed by *P. pseudoannulata* was recorded after 24 h. The experiment was conducted in a climatic chamber (28±2°C, 80±5% RH and L∶D 16∶8 h photoperiod).

### Laboratory spider feeding trial

Using a PCR assay, a laboratory feeding trial was conducted to determine how long *N. lugens* could be detected after spiders consumed them. Seventy-three healthy *P. pseudoannulata* adults of similar size were collected from the Experiment Farm of Zhejiang University and starved for 15 days, and thee spiders were collected as negative controls. Each spider was fed one female *N. lugens*. Following prey consumption, 10 spiders were collected for 0, 12, 24, 36, 48, 60 or 72 h and frozen at −70°C until assayed using the PCR technique describe below. Trials were conducted in a climatic chamber (28±2°C, 80±5% RH and L∶D 16∶8 h photoperiod). It should be noted that a similar feeding trail assayed using monoclonal antibody (McAb)-based ELISA had been conducted [13].

### Experimental Design and Field Management

The experiments were conducted during 2009 at the Experiment Farm of Zhejiang University (120.12°E, 30.13°N). Rice seeds of KMD1, KMD2 and Xiushui 11 were sown on 20 June, and the seedlings transplanted on 20 July. Four months after transplantation, the rice reached full maturity. The field was divided into nine experimental plots in a 3 (treatments, two transgenic rice lines and one non)×3 (replications) completely randomized design. Each experimental plot measured 20 by 25 m and was bordered on all sides by a 50-cm-wide unplanted walkway. Seedlings were hand transplanted at one seedling per hill spaced 16.5 by 16.5 cm apart, and the entire experimental field was surrounded by five border rows of non-transgenic control plants. Normal cultural practices, such as fertilization and irrigation, for growing rice were followed during the course of the experiment except that no insecticide was applied after sowing and transplanting.

### Field samples

On each sampling date, 7 October, 21 October and 5 November, 60 *P. pseudoannulata* adults (30 for PCR assay and 30 for ELISA assay) were collected from each plot respectively, transferred to 1.5 ml Eppendorf tubes and frozen at −70°C. Gut content of spiders were assayed using ELISA and PCR, as described below.

### PCR assay

#### DNA extraction

Prior to extraction, each spider was allowed to thaw at room temperature and its gut content was extracted. DNA was extracted using the Dneasy Tissue Kit (QIAGEN, Shanghai, China) following the manufacturer's instructions. For maximum yield, total DNA was eluted twice in the AE buffer provided by the manufacturer and stored at 4°C.

#### PCR amplification

We designed a *N. lugens*-specific primer set toward the mtDNA *COI* gene fragment. The primers were designed from the accession number HM160119- HM160160 (including 16 species commonly found in a rice paddy) reported in GenBank (forward 5′- TTATTAATTTCAAGATCACTAAC-3′; reverse 5′- GTCTCCACCCCCGGTG-3′; 351-bp). DNA applications were performed in a 25-µl reaction volume containing 1 µl of DNA extract, 0.5 µl of 10 mM deoxynucleotide triphosphates, 1 µl of each primer (5 µM), 1 U of *Tag* DNA Ploymerase, and 2.5 µl of 10× PCR buffer using a final MgCl_2_ concentration of 2.0 mM. PCR cycling conditions were as follows: 95°C (3 min) followed by 35 cycles at 95°C (30 s), 52°C (30 s) and 72°C (30 s), and then 1 cycle of 72°C for 10 min. PCR products were separated by electrophoresis in 1% agarose gel (100 V, 30 min). Each gel was stained with ethidium bromide, and the bands on the gel were visualized using BioSpectrum 410 Imaging System (UVP, Upland, CA). Samples were recorded as positive when a band appeared on the 351-bp region of the gel.

### ELISA assay

Field samples of *P. pseudoannulata* were screened using a *N. lugens*-specific sandwich ELISA to determine whether they preyed on *N. lugens*. This technique was described in detail by Zhao et al. [34]. Briefly, each spider was homogenized using a sterile pestle in 1 ml of phosphate buffer saline (PBS, pH 7.4). The homogenates were then centrifuged (4,500 g) at 4°C for 15 min. The supernatant diluted 1∶100 in PBS was used as the spider sample. The wells of a 96-well microplate were coated with 100 µl of the primary McAb 4B8 [34] diluted 1∶1,000 in carbonate buffer. After 2 h at 37°C, the primary antibody was discarded and 100 µl of blocking buffer (1% non-fat dry milk in PBS) was added to each well for 30 min. The blocking buffer was then discarded, rinsed three times with PBS-Tween 20 (0.05%) (PBST), and each well was coated with 100 µl of spider sample. After 1 h at 37°C, the samples were discarded and wells were rinsed as above. A 100 µl aliquot of the secondary McAb 4B8 conjugated to horseradish peroxidase [34] diluted 1∶4,000 in blocking buffer was added to each well and incubated for 1 h at 37°C. Plates were rinsed three times with PBST and twice wtih PBS. 100 µl of TMB substrate solution was then added to each well and incubated for 20 min at 37°C. The reaction was stopped by adding 50 µl of 3 M sulfuric acid to each well. Spectrophotometric measurements were taken using a multi-detection microplate reader (Synergy HT, Bio-Tek, Winooski, VT) at 450 nm. Each 96-well plate contained the following controls: (1) 6 PBS blanks, (2) a positive control (100 µl of 1 mg *N. lugens* in 1 ml PBS), (3) 8 individual negative spider controls (spider not fed *N. lugens*) and (4) a positive spider control (a spider fed a *N. lugens* female adult). Spiders were scored as positive if they yielded an ELISA response three standard deviations above that of negative spider control.

### Statistical analysis

ELISA data were analyzed using one-way analysis of variance [35] and Tukey's multiple-range test. Survival analyses of *P. pseudoannulata* were conducted using the Wilcoxon test for homogeneity. Data on the development time and reproduction of *P. pseudoannulata* fed on *Bt* and non-*Bt* rice-reared *N. lugens* were analyzed using one-way ANOVA and Tukey's multiple-range test. Data from the function response experiment were fitted to Holling's “Type II” disc equation [19]: *y = aTx/*(*1+abx*), where *y* is the number of prey attacked, *x* is the prey density, *T* is the time period of the bioassay (24 h). The parameters *a* (instantaneous search rate) and *b* (time required to handle a prey) were calculated using least-squares non-linear regression based on the Gauss-Newton method. Data on percentage of spiders from *Bt* rice or non-*Bt* rice fields testing positive for *N. lugens* remains were analyzed using repeated-measured ANOVA and Tukey's multiple-range test. All statistical calculations were performed with SAS version 9.1 package [36]. For all tests *α* = 0.05.
